# Efficacy of neuromodulation and rehabilitation approaches on pain relief in patients with spinal cord injury: a systematic review and meta-analysis

**DOI:** 10.1007/s10072-025-08077-y

**Published:** 2025-03-11

**Authors:** Simona Portaro, Angelo Alito, Giulia Leonardi, Nicola Marotta, Adriana Tisano, Daniele Bruschetta, Umile Giuseppe Longo, Antonio Ammendolia, Demetrio Milardi, Alessandro de Sire

**Affiliations:** 1 Department of Physical and Rehabilitation Medicine, University Hospital “G. Martino”, Messina, Italy; 2 Department of Biomedical, Dental Sciences and Morphological and Functional Images, University of Messina, Messina, Italy; 3 Physical Medicine and Rehabilitation Unit, Department of Experimental and Clinical Medicine, University of Catanzaro “Magna Graecia”, Catanzaro, Italy; 4 Research Center on Musculoskeletal Health, MusculoSkeletalHealth@UMG, University of Catanzaro “Magna Graecia”, Catanzaro, Italy; 5 Department of Clinical and Experimental Medicine, University of Messina, Messina, Italy; 6 Fondazione Policlinico Universitario Campus Bio-Medico, Rome, Italy; 7 Research Unit of Orthopaedic and Trauma Surgery, Department of Medicine and Surgery, Università Campus Bio-Medico Di Roma, Rome, Italy; 8 Physical Medicine and Rehabilitation Unit, Department of Medical and Surgical Sciences, University of Catanzaro “Magna Graecia”, Catanzaro, Italy

**Keywords:** Spinal cord injury, Pain, Rehabilitation, Non-invasive brain stimulation

## Abstract

**Introduction:**

Spinal cord injury (SCI) is a debilitating neurological condition that causes physical dependency, psychological distress, and financial burden. Pain is a common consequence of SCI, significantly impacting quality of life. Effective pain management in SCI is challenging and requires multifaceted approaches. Among rehabilitation methods, non-invasive brain stimulation techniques such as repetitive transcranial magnetic stimulation (rTMS), theta burst stimulation (TBS), transcranial direct current stimulation (tDCS), transcutaneous electrical nerve stimulation (TENS), and virtual reality (VR) have been explored. This study aims to evaluate the efficacy of rehabilitation and non-invasive brain stimulation techniques on pain relief in SCI patients.

**Methods:**

A systematic review of the literature was conducted using PubMed, Scopus, and ScienceDirect with the formula ("spinal cord injury") AND ("pain"). Risk of bias was assessed using the Cochrane Risk of Bias Tool.

**Results:**

Sixteen studies involving 319 patients were included. Patients in the control groups received: rTMS in seven trials, tDCS in seven trials, TBS in one trial, and TENS combined with VR in one trial. The trials analyzed were of poor methodological quality, characterized by small sample sizes, weak power analyses, varying clinical scores, and non-comparable follow-up periods. No major complications or serious adverse events were reported.

**Conclusion:**

Results were inconsistent, with no solid evidence supporting the superiority of rehabilitation techniques over comparator treatments. However, the favorable safety profile and positive outcomes in some measures suggest potential benefits for pain management and quality of life. Further studies are necessary to better understand SCI-related pain and optimize treatment strategies.

## Introduction

Spinal cord injury (SCI) is a devastating neurological condition causing physical dependency, morbidity, psychological stress, and financial burden [1]. SCI refers to damage to the spinal cord, extending from the foramen magnum to the cauda equina, and often results in significant disability for the affected individuals [1]. Every year, approximately 40 million people, mainly men aged 20–35, suffer from SCI with a global incidence of traumatic SCI of 22,5 new cases/million per year [2]. SCI prevalence has increased among the last 30 years, with estimated global rate between 250,000 and 500,000 individuals every year [3]. Road traffic accidents, gunshot and knife wounds, falls and sports injuries are the most common causes of SCI worldwide [4, 5]. SCIs are classified by the American Spinal Injury Association (ASIA) as "complete" or "incomplete" based on sacral sparing, considering motor and sensory functions [6].

The neurological level is defined as the lowest segment with normal function. ASIA A indicates a complete spinal cord injury (SCI), where there is no motor or sensory function preserved in the sacral segments S4-S5; ASIA B refers to a sensory-incomplete SCI; and ASIA C–D categorize motor-incomplete SCI [7]. Regardless of whether the injury is complete or incomplete, SCI rehabilitation and management are lifelong processes due to various complications, including neurogenic bladder and bowel, pressure ulcers, orthostatic hypotension, deep vein thrombosis, spasticity, autonomic dysreflexia, depression, and pain [8, 9].

SCI can lead to musculoskeletal sequelae, including volumetric muscle loss (VML) in a significant proportion of patients. VML following SCI is responsible for delayed recovery with negative consequences in terms of functional outcomes and additional changes in muscle tissue. These changes, such as increased intramuscular fat and a shift in muscle fiber composition (e.g., from type I to type IIA/X fibers), reflect the complex interplay of atrophy and remodeling processes following SCI. Emerging evidence highlights the intricate crosstalk between bone and muscle in these patients, suggesting common pathways of degeneration and repair [10–12].

SCI can lead to a variety of different types of pain, which can significantly affect an individual’s quality of life, and this pain can be classified into three broad categories: **nociceptive, musculoskeletal,** and **neuropathic pain.** The International Spinal Cord Injury Pain (ISCIP) Classification include different type of pain, both related and unrelated to the SCI, and it is divided into the following three tiers [13] i) pain type (i.e., nociceptive, neuropathic, other pain [e.g., fibromyalgia], and unknown pain [if pain type cannot be determined]); ii) pain subtype; iii) primary pain source (e.g., spasms-related pain or spinal cord compression) if known [14].

Neuroplasticity is an essential feature in the spontaneous recovery from an SCI, but it may produce negative consequences such as neuropathic pain, spasticity, and autonomic [15].

Neuropathic pain in SCI is considered one of the most debilitating and resistant treatment pain [16–18]. Such type of pain is caused by damage or dysfunction of the spinal cord that affects the transmission of pain signals and affects approximately 40% of patients after SCI and consists of spontaneous numbness, tingling/shooting or burning pain that interferes with the patient's function, participation and quality of life [19, 20].

Specifically, two types of pain are observed in SCI patients: the first type—radicular pain—occurs in a dermatomal distribution at the level of the injury (referred to as at-level pain), and the second type—central pain—occurs in a more diffuse distribution below the level of the injury (referred to as below-level pain) [21]. Although both types of neuropathic pain are severe and persistent, there may be some differences in the underlying mechanisms [22]. However, knowledge of the underlying mechanisms of SCI pain is unsatisfactory, making the treatment of this neuropathic pain a major therapeutic challenge [20].

It is known that pain perception in SCI is a complex process modulated by multifactorial causes. It has been reported that the injured spinal somatosensory circuitry may generate abnormal nociceptive signals, which the brain interprets as pain [23, 24]. Additionally, thalamic integrative circuits may act as both generators and amplifiers of nociceptive signals [25].

Sensory deafferentation following SCI leads to profound and lasting reorganization of cortical and subcortical sensory maps in the adult brain [26, 27]. The pathophysiological consequences of this cortical plasticity may contribute to the development of pain [27, 28]. Furthermore, among SCI patients, pain has been linked to elevated levels of stress, anxiety, and depression [29]. The negative impact of pain on psychological well-being may be due to physical changes caused by SCI, which alter the pain threshold and exacerbate pain symptoms [30]. Prolonged pain can increase psychophysiological stress and tension, further contributing to emotional distress and changes in mood [31].

Adequate pain management is strictly related to an accurate pain assessment. Pain intensity scales are pivotal in assessing the efficacy of interventions and are considered primary outcome measures in both clinical and research settings [32, 33]. The most used measures of pain intensity, even in SCI, are the Faces Pain Scale-Revised (FPS-R), Verbal Rating Scale (VRS), Numeric Pain Rating Scale (NPRS), and Visual Analogue Scale (VAS) [34]. These four scales properly identify pain modifications and they are reliable and reproducible over time [35]. Once assessed pain intensity, pain management in SCI is therefore complex and requires a tailored, multifaceted approach. Firstly, a pharmacological approach is often recommended for the treatment of neuropathic pain in SCI, although this has limited efficacy and several side effects, limiting patient compliance [36]. Indeed, patients with SCI and pain have often tried non-pharmacological treatments to manage pain [37, 38].

Among these strategies, early rehabilitation is essential to prevent complications of hypomotility and has also been shown to be effective in the treatment of neuropathic pain [19, 39, 40]. However, other valuable alternative therapeutic approaches to neuropathic pain— such as relaxation, massage and acupuncture— have been considered [41, 42], as have strategies aimed at reversing or modulating somatosensory neural reorganization after injury [27]. Among such techniques, non-invasive brain stimulation methods, such as repetitive transcranial magnetic stimulation (TMS) [43–45], theta-burst stimulation (TBS) [46], or transcranial direct current stimulation (tDCS) [47], transcutaneous electrical nerve stimulation (TENS) [48], neurofeedback training [49], the use of movement imagery [50, 51], mirror therapy [52] or ‘virtual’ mirror therapy [53] have been applied over the years.

Considering that SCI treatment is a continuous, lifelong, and strenuous process, burdened by the pain component, the rehabilitation plays a key role in the improvement of functioning (where possible) and quality of life [54–56].

However, to date there is still a lack of evidence on the specific rehabilitative approaches that can be used in the management of pain in SCI patients, and the knowledge of the nature, origin and mechanism of SCI-related pain could be crucial for choosing the appropriate treatment.

Therefore, the current systematic review of randomized controlled trials (RCTs) with meta-analysis aimed at synthesizing the existing evidence on the efficacy of rehabilitation approaches on pain relief in SCI patients to highlight how these techniques could reshape rehabilitation practice, improving pain management and quality of life of SCI patients.

## Methods

### Search strategy

A systematic review of the literature was conducted to search for English-language articles published from inception to 14th December 2024), according to the Preferred Reporting Items for Systematic Reviews and Meta-Analyses (PRISMA). The electronic databases PubMed, Scopus and Science Direct were searched using the following formula ("spinal cord injury") AND ("pain"). The present systematic review was registered on the International Prospective Register of Systematic Reviews (PROSPERO) with number: CRD42024595133.

### Selection criteria

After removing duplicates, two reviewers independently screened all the articles for eligibility in the systematic review. In case of disagreement, a resolution was found through consultation with a third reviewer to achieve a consensus. Articles were deemed eligible if they addressed the inquiries formulated according to the following PICO (Population, Intervention, Comparison, Outcome):

- P) Participants: patients with diagnosis of SCI.
- I) Intervention: neuromodulation and all the rehabilitative techniques considered as conventional rehabilitation approaches.
- C) Comparator: placebo/sham treatments.
- O) Outcome measure: pain assessment, using visual analogue scale (VAS), numeric rating scale (NRS), and verbal rating scale (VRS).

Inclusion criteria were: (1) RCT study design, (2) English language, (3) from indexed journals in the last 20 years (2004–14/12/2024), and (4) use of rehabilitation techniques for the management of pain in SCI patients. Exclusion criteria were: (1) studies on animals; (2) in-vitro studies; (3) papers written in languages other than English; (4) data that did not address the efficacy of rehabilitation interventions in reducing pain in SCI patients.

### Data extraction

Two independent reviewers systematically extracted data from the included studies using a tailored data extraction template within Microsoft Excel. In case of disagreement, resolution was attained through consultation with a third reviewer to establish consensus.

Relevant data were then abstracted into a database with the consensus of the two observers on (1) study design, (2) type of SCI, (3) sample size and patient characteristics, (4) outcome measures, (5) therapeutic protocol and follow-up, (6) summary of clinical outcomes, and (7) overall performance. The authors considered whether the RCTs showed adverse effects. The primary outcome of this review was the analysis of patient-reported subjective scores and pain at baseline and at the end of the intervention. All the findings were provided as a summary through the manuscript text and tables, to warrant an adequate explanation of the main findings of the included RCTs.

### Data synthesis and study quality

Risk of bias was assessed using the Cochrane Risk of Bias Tool for RCTs. The results were then converted to Agency for Healthcare Research and Quality (AHRQ) standards, which ultimately classify RCTs as "good quality", "fair quality" or "poor quality" as follows: "good quality": all criteria met (i.e., low for each domain); "fair quality": one criterion not met (i.e., high risk of bias for one domain) or two criteria unclear and assessment that this is unlikely to have affected the outcome and there is no known important limitation that could invalidate the results; and "poor quality": one criterion not met (i.e., One criterion not met (i.e. high risk of bias for one domain) or two criteria unclear and assessment that this was likely to have biased the outcome and there are important limitations that could invalidate the results; two or more criteria listed as high or unclear risk of bias.

### Statistical analysis

A pairwise meta-analysis was performed to combine the effects and assess the effectiveness of different treatments in relieving pain in spinal cord injury patients with neuropathic pain. The effect of treatment on pain was assessed for each paper by calculating the mean difference (MD) and associated standard deviation (SD). For trials that only provided median and interquartile range data, we calculated the standardized mean difference (SMD) and its standard deviation (SD) using Hozo [57]; This method was used to tackle the differences in interventions and outcomes across the studies. The variability among the comparisons was assessed using the Chi-squared and I^2^ tests. A value of I^2^ greater than 0.5 indicated significant heterogeneity among the articles. The mean difference was utilized as a measure of effect size (ES), and a random-effects model was employed to calculate the pooled estimates along with 95% confidence intervals (95% CI). All statistical analyses were carried out using Stata (version 16.0, StataCorp LP, College Station, Texas, USA).

## Results

### Study characteristics

**Fig. 1:**
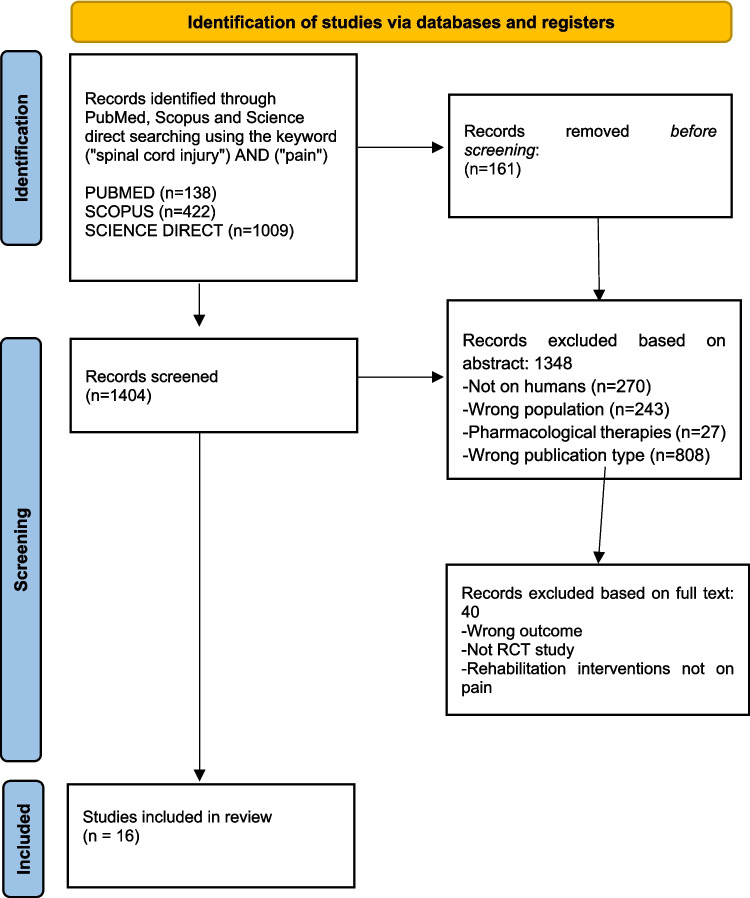
PRISMA flow diagram

The search of the scientific literature in the databases PubMed, Scopus and Science Direct yielded a total of 1569 potentially relevant publications. 161 duplicates were excluded from the search prior to screening. After reading the titles and abstracts, 1348 articles were discarded. After the full-text screening of the remaining 56 articles, 40 were excluded. Thus, the remaining 16 articles were included in the systematic review, as illustrated by the PRISMA 2020 Flow diagram (Fig. 1).

The pooled sample of these studies was a total of 319 patients affected by SCI. The studies' designs were extremely variable, since patients in the control groups received different injections or treatments: rTMS in seven studies, tDCS in seven studies, TBS in one study and TENS combined with VI in one study.

Table 1 provides an overview of the included studies and a summary of the results.

**Table 1:** Main findings of the included studies

Author	Study design	Patients features	Type of SCI	Intervention	Pain Features	Outcome	Assessment and Follow-up	Results	Overall performance	Side effects
Yeh, 2021	Double-blinded randomized controlled pilot trial	12 (6 vs 6) Sex not reported 48 (40.5–55.5) y in the EG 53 (37.3–62) y in CG	ASIA A: 4 patients (2 of EG; 2 of CG) ASIA B: 1 patients of EG ASIA C: 2 patients (1 of EG; 1 of CG ASIA D: 5 patients (2 of EG; 3 of CG)	12 individuals with NP after SCI were randomized into the EG or CG tDCS group. All participants received 12 sessions of real or sham tDCS, and moderate upper body exercises over 4–6 weeks. The real (or sham) stimulation were administered for 20 min before upper body exercise training	Left for more painful side: 5 patients Right for more painful side: 4 patients Symmetry of pain: 3 patients NP was diagnosed by a physiatrist according to the definition, “the pain arising as a direct consequence of a lesion or disease affecting the somatosensory system,” for more than 6 months with NRS ≥ 4/10	NRS 0–10; Self-questionnaire Neuropathic pain symptom inventory	The pain intensity was assessed by a 0–10 NRS in the last 24 h. All outcomes were assessed on the next day after the last session of the intervention for the post-test assessment. The follow-up assessment was taken on the day after 4 weeks since the post-test assessment	The multiple sessions of anodal tDCS combined with moderate upper body exercise were feasible for people with SCI and chronic NP but the analgesic effect was not superior to exercise alone after 12 sessions of intervention, and the beneficial effect was observed at 4-week follow-up	tDCS =	One participant reported mild headache and dizziness, and three reported tingling feeling over the stimulated sites during real tDCS application. These symptoms subsided once the tDCS completed There were two participants with erythematous rash underneath the electrode, which subsided within an hour
Zhao et al., 2020	Randomized, double-blinded, two-group parallel clinical trial	48 participants 41.6 ± 9 years	37 complete SCI 11 partial SCI occurring less than three months before	Patients randomly assigned to the rTMS (10 Hz, 1,500 stimuli) or sham group. Besides rTMS, conventional physical therapy, occupational therapy and medical treatments were provided to all patients	Central neuropathic pain	NRS, Short-Form McGill Pain Questionnaire-2	The assessments were carried out at baseline (T0), three days (T1), one week (T2), two weeks (T3), and three weeks (T4) after onset of treatment	10 Hz rTMS over the hand area of the motor cortex could alleviate acute central neuropathic pain in the early phase of SCI	Significant reduction in pain in the rTMS group. In contrast, there were no significant within-subject effects in the sham group	4 patients reported slightly uncomfortable but reversible periods of numbness in the scalp and twitching of facial muscles during rTMS, but no serious adverse effects were observed
Choi, 2019	A single-blind crossover study	10 (7 M) 20 years ≤ age < 90 years	Cervical complete motor SCI, at least 6 months after SCI ASIA A: 6 patients ASIA B: 4 patients	The effect of single sessions of both anodal and sham tsDCS on chronic NP in 10 volunteers with complete motor cervical SCI was assessed	Abdomen: 1 patient Both buttock and hip: 1 patient Both lower legs and feet: 4 patients Lower back: 1 patient Both shoulder and arm: 2 patients Both upper leg/thigh: 1 patient Continuous chronic pain for at least the 3 preceding months and a score ≥ 4 on VAS at the baseline/start of the treatment, and refractoriness to drugs for pain relief, such as antidepressant, antiepileptics, and/or opioids	NRS, PGA, PPI	Pre- to post-tsDCS intervention changes in pain intensity, patient global assessment, and present pain intensity, were assessed before and after the tsDCS session (immediately post stimulation, and at 1 and 2 h post stimulation)	No significant pre- to post-treatment difference in pain intensity between the active and sham tsDCS groups. Only in the sham tsDCS stimulation, NRS was reduced after the stimulation session. A single session of anodal tsDCS is feasible but does not have a significant analgesic effect in individuals with chronic cervical SCI	tsDCS =	Most participants felt tingling at the anodal or cathodal site, the tingling sensation was transient and tolerated
Sun, 2019	Double-blind, sham-controlled, clinical trial	17 (11 vs 6) rTMS group: 11 patients Sham group: 6 patients 15 M 37.00 (23.00–54.50) y	Complete or incomplete SCI ASIA A: 12 patients ASIA C: 3 patients ASIA D: 2 patients	Right-handed patients with NP after SCI were randomized to receive a session of rTMS daily for 6 weeks with a one-day interval per week. The CG received sham treatment over the left primary motor cortex week corresponding to the hand area	NP at or below the lesion level with an average pain intensity, as assessed by NRS, of no less than 4 at baseline and before TMS treatment Bilateral lower limbs: 7 patients Back: 4 patients Left shoulder, Thoracic, Back: 1 patient Trunk: 1 patient Abdomen: 1 patient Limbs, Thoracic, Abdomen: 1 patient Shoulders, arms, hands: 1 patient Limbs, Trunk: 1 patient	NRS	The NRS was assessed and fNIRS was performed at baseline (T0), after the first session rTMS (day 1, T1), and at the end of week 1 (T2), 2 (T3), 4 (T4), and 6 (T5)	The pain intensity gradually decreased over time in both the rTMS group and sham groups. High-frequency rTMS treatment over the hand area of the left M1 for more than 2 weeks effectively enhances the analgesic effects of conventional rehabilitation and pharmaco-logical therapies on NP after SCI, which may partially result from the amelioration of M1 and PMC hypersensitivity	rTMS +	Not reported
Gharooni, 2018	Single-blind, sham-controlled, crossover randomized feasibility study	10 (8 M) Active rTMS group: 10 patients Sham rTMS group: 10 patients 46.8 ± (11.9) y	Participants with incomplete cervical SCI sustained at least 3 months ago ASIA A: 0 patients ASIA B: 1 patient ASIA C: 4 patients ASIA D: 5 patients	Participants with incomplete SCI and suffering with upper-limb spasticity were randomized to receive active/sham iTBS over the hand representation of the PMC. The intervention was delivered in 10 sessions over a 2-week period, followed by a 2-week washout, before being crossed over to receive the alternative intervention for the same number of sessions	Not reported A major limitation is that participants were not recruited based on their level or type of pain	VAS-pain 0–100**,** VAS spasticity**,** MAS, LASIS	Outcomes assessments were performed at baseline and at the end of intervention. A follow-up visit was performed at 2 weeks post intervention	Effects on sensorimotor function and pain indicate that the intervention protocol is unlikely to lead to any significant improvements in ASIA and VAS pain outcomes	iTBS =	One participant reported interscapular “tightness” the morning after their first session (active). It was unrelated to the intervention as the participant had similar experiences prior to enrollment which was attributed to ongoing spasticity
Nardone et al., 2017	RCT Active rTMS vs sham	Active rTMS (n = 6, 43,67 ± 11,57); sham (n = 6, 42,5 ± 13,59)	Active rTMS: ASIA A: 1, ASIA B: 2, ASIA C: 1, ASIA D: 2 (C5-T8); time after SCI: 9,83y Sham: ASIA A: 1, ASIA B: 3, ASIA C: 1, ASIA D: 1 (C6-T10); time after SCI: 10,67 y	rTMS of the PMC/DLPFC in subjects with SCI and neuropathic pain	Active rTMS baseline pain: VAS 6,66; affective pain MPQ 21,66; sensory pain MPQ 8. Sham baseline pain: VAS 6,83; affective pain MPQ 22,33; sensory pain MPQ 7,83	VAS, MPQ	Baseline (T0), 1 day after the first week of treatment (T1), 1 day (T2), 1 week (T3) and 1 month (T4) after the last intervention	Subjects who received active rTMS had a statistically significant reduction in pain symptoms compared to their baseline pain, whereas sham rTMS participants had a non-significant change in daily pain compared to their baseline pain	rTMS +	Two subjects undergoing active rTMS reported slightly unconfortable twiching of facial muscles during rTMS
Thibaut et al., 2017	two-phase randomized sham-controlled clinical trial	Patients with sublesional neuropathic pain (VAS ≥ 4). 33 in Phase I, active tDCS (n = 16, 51.38 ± 14.89y), sham tDCS (n = 17, 51.00 ± 10.11). 9 in Phase II, active tDCS (n = 9, 49.47 ± 16.01), sham tDCS (n = 3,47.67 ± 3.54)	Time after SCI: active tDCS 5.81 ± 6.27y; sham 4.56 ± 3.54	5 days of tDCS followed by a 3-month follow-up period (Phase I). Phase II consisted of 10 days of tDCS with an 8-week follow-up period	Baseline pain intensity (VAS): active tDCS 5.81 (± 6.27); sham 4.56 (± 3.54)	VAS pain	Phase I: baseline, at the end of the 5 stimulation sessions, at 1-week and 3-month follow-up Phase II: after 5 and 10 stimulation sessions and at 2-, 4- and 8-weeks follow-up	The treatment effect was observed at the 1-week follow-up in Phase I and at the 4-week follow-up in Phase II. The overall pain level was significantly lower in the active group compared to the sham group in Phase II. tDCS appears to be a promising tool for pain management in SCI patients, and repeated stimulation sessions are needed to induce long-lasting effects. It appears that the addition of a second treatment period could induce long-lasting effects	tDCS +	The majority reported mild to moderate tingling or itching during both active and sham during both active and sham stimulation. No unexpected adverse effects were observed
Ngernyam et al., 2015	Randomized double-blind controlled placebo cross-over trial active vs sham tDCS	20 (15 M) 44.5 + 9.16y	ASIA A: 9, ASIA B: 3, ASIA C: 6, ASIA D: 2 (C3-T11). Time after SCI: 54.65 ± 38.66	Participants were randomised to receive either active tDCS followed by sham tDCS or sham tDCS stimulation followed by active tDCS in a 1:1 ratio	Pain at and below level: 5; pain below level 15. Duration of pain: 50,10 ± 37,06 m. Baseline pain (NRS): 5,77 ± 1,69	NRS	(1) 1 week of baseline assessment; (2) a single session of 2 mA of active or sham tDCS (depending on the order) for 20 min; (3) 1 week of assessment and washout; (4) another week of baseline assessment; (5) another session of active or sham tDCS (depending on the order); and (5) an outcome assessment at 1 week	The active treatment condition but not sham treatment resulted in significant decreases in pain intensity	Active tDCS +	7 participants evidenced erythematous rash under the cathode (negative) electrode in the active tDCS condition
Özkul et al., 2015	Randomised controlled cross-over trial VI and TENS	24 (18 M) in two groups, 32.33 ± 12.97 with neuropathic pain	ASIA A: 17, ASIA B: 4, ASIA C: 3, (C5-L2). Time after SCI: 18,96 m	VI or TENS for 2 weeks, 1 week of wash out and then the other treatment	Pain duration: 12.46 ± 17.83 m. Pain sites: back: 3, lower limb: 2, knee: 4, calf: 3, ankle: 24, foot: 15	VAS, MPQ, NPS, BPI	Assessments were performed a total of four times, before and after both treatment applications	Pain intensity decreased immediately after both applications. After TENS application for 2 weeks, most and least pain intensity decreased significantly, while there was no significant decrease in pain intensity after 2 weeks for VI	VI, TENS +	Not reported
Yilmaz et al., 2014	Single center, prospective, randomized, double-blinded, controlled study Real rTMS versus sham rTMS	16 (16 M) real rTMS (n = 9) (40.0 ± 5.1y) vs sham rTMS (n = 7) (36.94 ± 8.0y)	ASIA Real rTMS: A = 4, B = 2, C = 1, D = 2 Complete: 5 ASIA Sham: A = 4, B = 2, C = 1 Complete: 5 Time after SCI: Real rTMS: 142.9 ± 92.8 m Sham 123.4 ± 101.5 m	rTMS for intractable neuropathic pain 1 treatment per day for 10 days	Upper and lower legs = 8; buttocks and both legs = 2; lower legs = 2; left upper leg and genitals: 1; genitals, upper and lower legs: 1; low upper legs and low lower leg: 1; genitals and upper legs: 1 Duration of pain: Real rTMS: 32.3 ± 25.9 m Sham 35.4 ± 17.9 m Baseline pain (VAS): Real rTMS: 7; Sham 7	VAS pain (0–10)	Baseline, after 10 days, 6 weeks, 6 months	Both real and sham rTMS produced a significant reduction in VAS scores. Post-hoc analysis showed that the significant difference was at 10 days and 6 weeks compared to baseline in the real rTMS group and only at 10 days compared to baseline in the sham rTMS group. The analgesic effect of rTMS on intractable neuropathic pain in SCI was not superior to placebo	rTMS =	Not reported
Jettè et al., 2013	Double-blind, cross-over randomized study	16 patients (11 M 5 F) 50 ± 9 y	16 SCI patients preserving hand muscle function 12 paraplegic 4 tetraplegic 8 had motor-complete SCI	Three single sessions of sham or active rTMS (10 Hz, total of 2000 stimuli) were applied in random order over the hand or leg area with a minimal 2-week interval	Neuropathic pain for at least 3 months, at or below SCI level	NRS, at 0 and 20 min postrTMS	Brain MRI prior to 2 active rTMS sessions (hand/leg M1 area) and 1 sham rTMS session in a randomized, counterbalanced order (at least 2 weeks apart)	Active rTMS applied to either the hand or the leg area, induced a significant reduction in pain for the first 48 h post intervention. Incomplete lesion showed greater analgesia than complete lesion	rTMS +	Not reported
Wrigley et al., 2013	RCT with crossover design Real tDCS vs sham	10 (8 M) (56.1 ± 14.9y)	Complete thoracic SCI Time after SCI: 21.3 ± 13.8y	For each active treatment, one 20-min session was delivered each day for 5 consecutive days Subjects were not requested to cease medications before the trial	Neuropathic pain below the neurological level of SCI Duration of pain: 15.8 ± 11.3 y Baseline pain (NPS): 5.6 ± 0.5	NPS (0–10)	Baseline, after each period of intervention, and at 4 weeks and 6 months	After 5 tDCS treatment sessions, mean pain intensity and unpleasantness ratings were not significantly different from baseline. tDCS did not provide pain relief, and a similar lack of effect was seen after sham treatment. The duration of the injury was quite long, and it is possible that tDCS is only an effective analgesic for relatively recent injuries and pain	tDCS =	Redness was reported by 80% of participants after active treatment and 90% of participants immediately after sham treatment on at least 1 of the 5 days. Tingling was reported by 80% of participants during active treatment and 70% during sham treatment. Fatigue was reported frequently for the active treatment (60%) but less frequently for the sham treatment (30%). Other side effects were less commonly reported but included dizziness (20% for active and sham treatment) and headache (10% for active and sham treatment). Most side effects were considered mild. The incidence of reported adverse events in the 24-h post-treatment period did not differ between the active and sham treatments
Soler et al., 2010	Randomized double blind, placebo-controlled study, parallel group design	40 (30 M) 45 ± 15.5 y	Complete or incomplete SCI with pain for at least 6 months following trauma or disease of the spinal cord	Patients were randomly assigned to receive: tDCS alone, visual illusion alone (in the mode of ‘virtual walking illusion’), or the combination of both interventions (tDCS + visual illusion)	Chronic neuropathic pain at or below SCI level for at least 6 months	NRS	5 evaluations: before treatment (baseline), at Day 14 (last day of treatment), at Day 24 (first follow-up) and at Day 38 (second follow-up) after initiation of treatment and 12 weeks after treatment (third follow-up)	The combination of tDCS and visual illusion reduced the intensity of neuropathic pain significantly more than any of the single interventions. The tDCS group showed improvement in continuous and paroxysmal pain. The visual illusion group improved only in continuous pain and dysaesthesias	tDCS combined with visual illusion +	Mild headache during active tDCS, tiredness, transient increase of neuropathic pain
Kang et al., 2009	Blinded, randomized crossover study	13 patients (6 M 5F) 54.8 ± 13.7 y	6 patients with paraplegia, 5 patients with tetraplegia, 5 patients had motor complete SCI, 6 had motor incomplete SCI	rTMS applied on the hand motor cortical area using a figure-of-eight coil. One thousand stimuli were applied daily on 5 consecutive days. Real and sham rTMS were separated by 12 weeks	Chronic neuropathic pain at multiple sites in the body, including the lower limbs, trunk, and pelvis	NRS, BPI	The NRS and BPI were measured before the first session, immediately after the third and fifth stimulation sessions, and 1, 3, 5, and 7 weeks after the end of the 5-day stimulation period	The changes in the NRS score for average pain over the preceding 24 h and the interference items of the BPI did not differ between the real and sham rTMS treatments, and thus, therapeutic efficacy of rTMS was not demonstrated	rTMS =	Not reported
Defrin et al., 2007	Double-blind randomized controlled trial	12 (7 M 4 F) paraplegic SCI patients 54 ± 6 y	Traumatic thoracic SCI, 2 complete SCI 9 incomplete SCI	Real or sham 10 daily motor rTMS treatments (500 trains at 5 Hz for 10 s; total of 500 pulses at intensity of 115% of motor threshold) using figure-of-8 coil over the Vertex	Chronic central pain	VAS, McGill Pain Questionnaire, Pain threshold, Beck Depression Inventory	Mean follow-up period was 4.5 weeks; follow-up period ranging from 2 to 6 weeks at the end of which MPQ assessment and BDI were obtained	Both real and sham TMS induced a significant reduction in VAS immediately after each of the 10 treatment sessions. However, SCI patients may benefit from a series of rTMS treatments	TMS =	Not reported
Fregni et al., 2006	3-week double-blinded treatment daily sham or active tDCS (2 mA, 20 min for 5 consecutive days) for 5 consecutive days	17 patients (14 M 3 F) 11 active tDCS 6 sham tDCS 35.7 ± 13.3 y	Traumatic spinal cord injury, due to fall, car accident or gun shot	tDCS – on pain control in patients with central pain due to traumatic SCI	Below lesion and in transition zone; stable chronic pain for at least the three preceding months	VAS, Beck Depression Inventory, Clinician Global Impression and Patient Global Assessment	All evaluations were performed by a blinded rater twice at baseline (2 weeks), daily during treatment period and once in the follow-up visit. Follow-up period: 16 days	Significant pain improvement after active anodal stimulation of the motor cortex, but not after sham stimulation	tDCS +	Not reported

*ASIA* American spinal injury association, *BCM* broad compression massage, *BPI-SF* brief pain inventory short form, *BTX-A* botulinum toxin type A, *CES* cranial electrotherapy stimulation, *CG* control group, *DAMP* deviation motor action potential, *DASS-21* depression anxiety stress scale, *EG* experimental group, *FBI* full body illusion, *fNIRS* functional near-infrared spectroscopy, *GI* guided imagery, *GPS* global pain scale, *HMDVR* head-mounted virtual-reality, *IPQ* iGroup presence questionnaire, *ISCIPBDS* SCI pain basic data set, *iTBS* intermittent theta-burst stimulation, *KT* kinesiotaping, *LASIS* leeds arm spasticity impact scale, *LCT* light contact touch, *LEMS* lower extremity motor score, *LT* locomotor training, *M1* primary motor cortex, *MAS* modified ashworth score, *MICT* moderate-intensity continuous training, *MPQ-BF* absorption scale of the multidimensional personality questionnaire – brief form, *MSK* musculoskeletal; *MT* massage therapy, *NP* neuropathic pain, *NPRS* numerical pain rating scale, *NPS* neuropathic pain scale, *NRS* numerical rating scale, *PGA* patient global assessment, *PMC/DLPFC* premotor cortex/dorsolateral prefrontal cortex, *PPI* present pain intensity, *QOL* quality of life questionnaire, *RAGT* robot-assisted gait training, *RCT* randomized controlled trial, *RPE* ratings of perceived exertion, *rTMS* repetitive transcranial magnetic stimulation, *SCS* spinal cord stimulation, *SCI* spinal cord injury, *SF-MPQ* short-form McGill pain questionnaire, *SS* sympathetic somatomotor, *ST* silk tape, *SIT* sprint interval training, *TCET* transcranial electrostimulation treatment, *tDCS* transcranial direct current stimulation, *TENS* transcutaneous electrical nerve stimulation, *VAS* visual analogue scale, *VI* visual illusion, *VLI* virtual leg illusion, *VR* virtual reality, + plus, = equal

### Quality assessment

Looking at the quality of the available literature by the AHRQ standard, we found that all the 16 RCTs included in this revision reached a “Poor quality”. The results of the analysis performed with the Cochrane Risk of Bias tool for RCT are detailed in Table 2. Eight of the 16 included studies were double-blind. The random sequence generation was specified in 7 studies [58–64], instead of the method of allocation concealment, which was described only in 3 studies [58, 61, 65]. In addition, the risk of attrition bias for the included RCTs was low for all the included studies, except for one trial [66]. Moreover, in 2 studies, it was clearly stated how many patients were screened, how many were excluded from randomization and why, and how many were lost to follow-up, specifying the reason [63, 67]. Flow diagrams showing the patient selection process were reported only in 7 of the 16 included RCTs [58, 63, 65–69]. Finally, we found that, except for two studies [58, 67], the protocol trials were not registered in a public registry, which should be mandatory according to the Consolidated Standards of Reporting Trials (CONSORT) 2010 guidelines.

**Table 2:** Quality assessment of the included studies by using the cochrane risk of bias tool for randomized controlled trials and the AHRQ (Agency for Healthcare Research and Quality) standards

Publication	Random sequence generation	Allocation concealment	Selective reporting	Other bias	Blinding of participants and personnel	Blinding of outcome assessment	Incomplete outcome data	AHRQ standard
Yeh et al., 2021	Unclear	Unclear	Unclear	Unclear	Low	High	Low	Poor
Zhao et al.,2020	Unclear	Unclear	Unclear	Unclear	Low	Low	Low	Poor
Choi et al., 2019	Low	Low	Unclear	Unclear	High	High	Low	Poor
Sun et al., 2019	Low	Unclear	Unclear	Unclear	Low	Low	Low	Poor
Gharooni et al., 2018	Unclear	Low	Unclear	Unclear	Low	High	Low	Poor
Nardone et al., 2017	Unclear	Unclear	Unclear	Unclear	Low	Low	Low	Poor
Thibaut et al., 2017	Unclear	Unclear	Unclear	Unclear	Low	Low	High	Poor
Ngernyam et al., 2015	Low	Unclear	Unclear	Unclear	Low	Low	Low	Poor
Özkul et al., 2015	Low	Low	Unclear	Unclear	Unclear	Unclear	Low	Poor
Yilmaz et al., 2014	Low	Unclear	Unclear	Unclear	Low	Low	Low	Poor
Jettè et al., 2013	Unclear	Unclear	Unclear	Unclear	Low	Low	Low	Poor
Wrigley et al., 2013	Unclear	Unclear	Unclear	Unclear	Low	Low	Low	Poor
Soler et al., 2010	Low	Unclear	Unclear	Unclear	Low	Low	Low	Poor
Kang et al., 2009	High	High	Unclear	Unclear	Unclear	Low	Low	Poor
Defrin et al., 2007	Unclear	Unclear	Unclear	Unclear	Low	Low	Low	Poor
Fregni et al., 2006	Low	Unclear	Unclear	Unclear	Low	Low	Low	Poor

“Good quality”: All criteria met (i.e., low for each domain); “Fair quality”: One criterion not met (i.e., high risk of bias for one domain) or 2 criteria, and the assessment that this was unlikely to have biased the outcome, and there is no known important limitation that could invalidate the results; “Poor quality”: One criterion not met (i.e., high risk of bias for one domain) or 2 criteria unclear, and the assessment that this was likely to have biased the outcome, and there are important limitations that could invalidate the results; Poor quality: two or more criteria listed as high or unclear risk of bias

### Meta-analysis

**Fig. 2:**
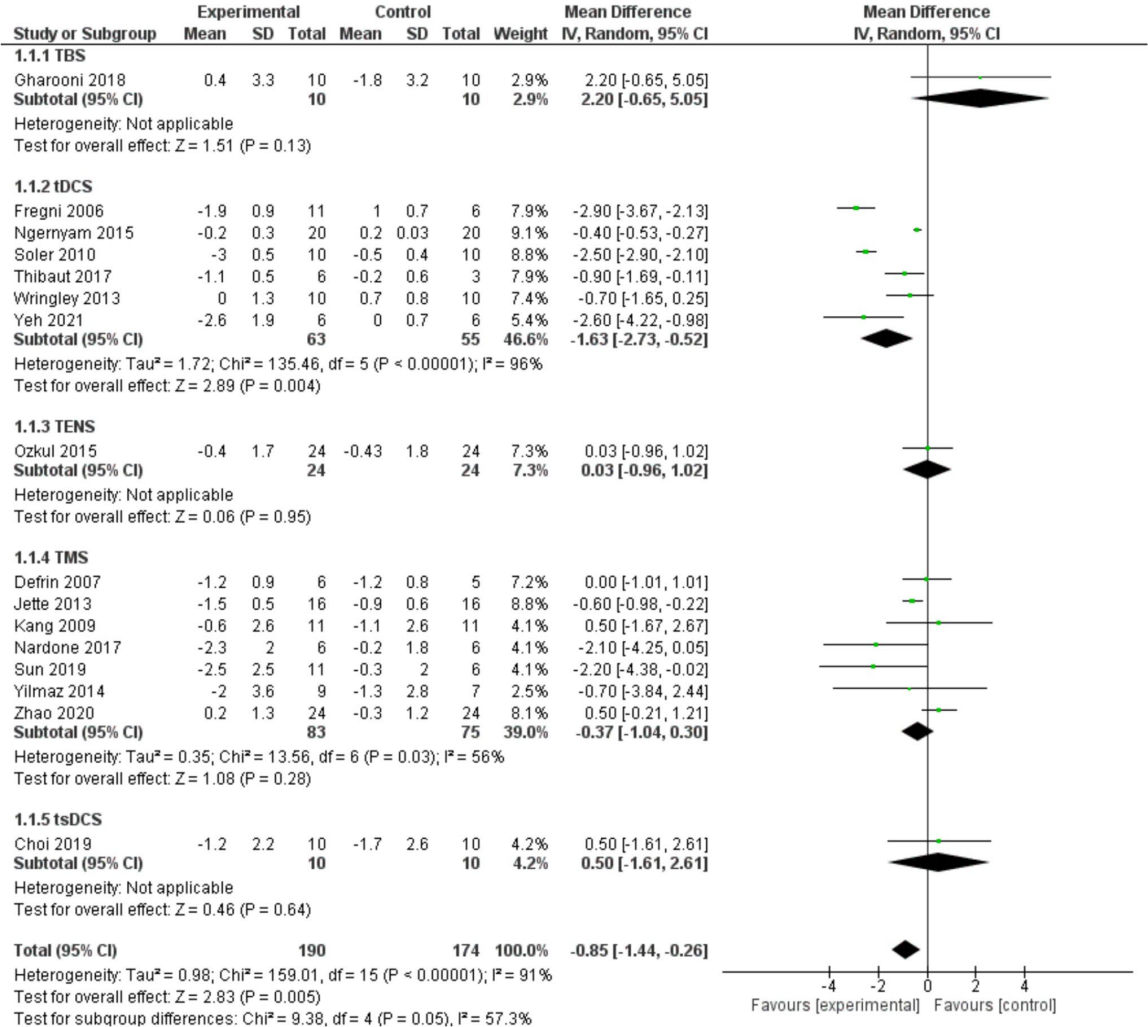
Pairwise meta-analysis to evaluate the effectiveness of non-invasive neuromodulation techniques and TENS combined with VI, aimed at alleviating pain (measured using VAS or NRS) in patients with SCI

A pairwise meta-analysis was conducted to evaluate the effectiveness of non-invasive neuromodulation techniques and TENS combined with VI, aimed at alleviating pain (measured using VAS or NRS) in patients with spinal cord injury (SCI). The results indicated that these rehabilitation methods yielded an overall effect size (ES) of −0.85 [−1.4, −0.3] (p = 0.0005) in reducing pain among these patients. However, significant heterogeneity was found across the studies (I^2^ = 91%, p < 0.0001), leading to the adoption of a random effects model. In the subgroup analysis, a notable effect size was only observed in the tDCS subgroup, which had an effect size of −1.63 [−2.73, −0.52] (p = 0.0001), as illustrated in Fig. 2.

## Discussion

To better define which rehabilitation approach should be classified as first line treatment in SCI pain management, it is imperative to identify the causes of pain and the mechanisms of action of the different rehabilitation techniques included in this study. Neuropathic pain can be spontaneous or triggered by various stimuli. It may be continuous or intermittent and is often accompanied by sensory disturbances, such as paresthesia (tingling) and dysesthesia (abnormal or unpleasant sensations) [70]. The causative mechanisms at the basis of neuropathic pain have been identified in the neuronal hyperexcitability seen after SCI and may be related to functional changes in receptors (i.e., N-methyl-D-aspartate [NMDA] and other glutamate receptors) and altered expression of ion channels in the spinal cord and thalamus [71]. Moreover, neurons and glia cells release inflammatory mediators, cytokines, chemokines, thus maintaining central pain [72–74]. The altered function of descending inhibitory and facilitatory pathways and loss of GABAergic inhibitory interneurons may contribute to neuronal hyperexcitability. In addition, after SCI, GABAergic function is also reduced by the downregulation of the potassium chloride exporter KCC2 and the increased function of NKCC1 at the epicenter of the lesion, which alter Cl- homeostasis, leading to sensory hypersensitivity [75]. It has also been suggested that the thalamus is a pain generator, as abnormal bursting activity has been recorded in the thalamus after SCI [76]. However, central pain also involves other areas of the brain, such as the prefrontal cortex and anterior cingulate cortex, but it remains to be established whether these cortical changes are pain mechanisms rather than effects of changes in subcortical areas [27, 70, 77].

Garcia-Larrea et al. proposed the ‘thalamus pain-related structure pathway’ as the substrate for pain relief induced by motor cortex stimulation [78].

Another possibility is the ectopic impulses generated in the damaged spinothalamic tract or other ascending tracts [70].

Understanding the underlying mechanisms of spinal cord injury (SCI)-related neuropathic pain is important for the development of new tailored medications, but despite the availability of several treatment options, most SCI patients do not achieve significant pain relief with these medications or discontinue treatment due to drug-related side effects [79–82].

On this basis, innovative rehabilitation approaches have been proposed over the years to relieve SCI-related neuropathic pain.

Among these techniques, non-invasive brain stimulation has been reported, although its efficacy is variable and still under debate. Understanding their mechanisms of action may help to select the best techniques, combined or not with medication, to better manage SCI-related chronic neuropathic pain and thus improve patients' quality of life. In this review, we mainly included RCTs discussing the role of tDCS, tsDCS, TMS and TBS.

tDCS is considered a purely neuromodulatory intervention, enhancing synaptic plasticity in response to combined inputs [83]. tDCS has been reported as a promising tool for the treatment of SCI-related neuropathic pain, through a combination of cortical and spinal mechanisms to modulate pain processing pathways, enhance descending inhibition, promote neuroplasticity, and reduce central sensitization [84]. Modulation of cortical excitability is one of the possible mechanisms of action of tDCS in SCI-related neuropathic pain, achieved by anodal and cathodal stimulation. tDCS over M1 induces activity changes in several brain regions, including the primary somatosensory cortex, somatosensory thalamus (ventroposterior nucleus), insular cortex, prefrontal cortex, and nucleus accumbens [85].

In addition, unilateral M1 tDCS has been shown to produce significant changes in activity in both the underlying M1 and M1 on the contralateral side of the brain. Although the mechanism by which M1 tDCS can relieve pain in people with SCI remains unknown, it is possible that the analgesic effects are mediated by its influence on activity in regions such as the somatosensory thalamus. It was reported over a decade ago that neuropathic SCI pain is associated with abnormal thalamic firing patterns [60, 62] and altered thalamocortical rhythm [86].

Anodal tDCS applies a constant electrical current to the primary motor cortex (M1) or sensory cortex to increase neuronal excitability and promote adaptive reorganization of the cortex, thereby reducing cortical representations of pain [84]. On the other hand, with cathodal stimulation, the cathodal electrode placed over areas such as the contralateral somatosensory cortex can reduce cortical excitability, which may help to reduce pain processing [87].

Another proposed mechanism of action of tDCS is the enhancement of the inhibitory signals that reduce pain transmission in the spinal cord descending [84]. Moreover, tDCS may modulate the balance between excitatory (glutamate) and inhibitory (GABA) neurotransmitter systems in both the brain and spinal cord [88].

tDCS has also a role in the reorganization of Pain Processing Networks, since it promotes neuroplastic changes in the brain to counteract the after SCI maladaptive cortical reorganization [84]. In addition, tDCS has been shown to activate brain areas such as the prefrontal cortex, anterior cingulate cortex, and insula, which are involved in the cognitive and emotional processing of pain areas [87].

tDCS even has a role in spinal cord modulation, modulating spinal cord activity and potentially lowering the threshold for pain perception [60].

On this basis, tDCS has been used in the field of SCI-related neuropathic pain, although the effects are variable. tDCS individual variability can be influenced by several factors, such as the level of injury, the time after injury, electrode placement, intensity, and duration of pain [60, 84, 87].

The role of tDCS in SCI-related neuropathic pain has been considered over the last 20 years. First, in 2006, Fregni et al. conducted a randomized controlled trial (RCT) to evaluate the effects of tDCS on pain management in individuals with traumatic spinal cord injury (SCI) and central pain. They reported a significant improvement in pain levels as measured by the Visual Analog Scale (VAS) [59]. The proposed mechanism behind the pain reduction observed in this study was related to anodal tDCS, which is thought to increase cortical excitability, with effects that persist beyond the duration of the stimulation [89, 90].

In 2010, Soler et al. evaluated the analgesic effect of tDCS of the motor cortex associated to techniques of walking visual illusion to treat after SCI neuropathic pain, demonstrating the intensity improvement of neuropathic pain, gained through the ‘thalamus pain-related structure pathway [62]. In the Soler study, the combination of both techniques (tDCS + visual illusion) increases corticospinal excitability beyond either observation or imagery alone [91] and leads to a greater reduction in intracortical inhibition [92].

On the other hand, anodal tDCS is associated with an increase of cortical excitability [89, 93]. In terms of the combined effect with visual illusion, moving images of paralyzed body parts in SCI patients increase activity in the same brain regions as in healthy people, including the primary motor cortex [94, 95].

Later, in 2013, Wrigley et al. raised the possibility that tDCS focused over M1 had an analgesic effect only in recent SCI, possibly because central changes were consolidated, so that tDCS did not act on the modulation of central pain-related circuits [68].

To better elucidate the effects of tDCS on neuropathic pain, Ngernyam et al. in 2015 demonstrated that anodal tDCS over M1 significantly reduced pain intensity via increased M1 cortical excitability, with increased intrahemispheric connectivity across multiple bandwidths (particularly in the theta-alpha range) [60]. Nevertheless, thalamic activity plays a central role in signal transmission, and influencing this activity by stimulating the motor cortex has been proposed by many researchers as a potential mechanism of tDCS [59, 62, 68, 96, 97].

These findings suggest that increased motor cortex activation induced by tDCS can potentially influence inhibitory neurons in both adjacent and distant brain areas [98], whereas an increase in frontal inhibitory systems may exert an actively control on pain perception [99]. Given the variable response of tDCS on SCI-related pain, other factors possibly influence tDCS response and effectiveness.

In 2017, Thibaut et al. assessed the immediate and long-term effects of tDCS on SCI-related neuropathic pain, and while they did not confirm the efficacy of tDCS as a promising tool for managing chronic neuropathic pain, they did state that repeated tDCS sessions for acute pain treatment may limit the pathological reorganization of the pain matrix that leads to the development of neuropathic pain [66]. In addition, the authors highlighted the influence of tDCS on sodium and calcium channel opening and NMDA receptor excitability, while long-lasting effects are analogous to activity-dependent synaptic plasticity, namely long-term potentiation (LTP) and long-term depression (LTD) [100, 101].

In 2019, Choi et al. evaluated neuropathic pain in SCI after application of transcutaneous spinal direct current stimulation (tsDCS) and showed no significant difference in pain intensity between the active and sham tsDCS groups before and after treatment. The lack of efficacy may be attributed to the different electrode placement used in comparison to standard tDCS protocols. In this case, the active electrode was positioned over the spinous process of the tenth thoracic vertebra, while the reference electrode was placed on the top of the head. This setup may have affected the overall effectiveness of the treatment [58].

tsDCS is a non-invasive method that, like tDCS, uses constant currents through skin patch electrodes [102]. Both tDCS and tsDCS induce a polarization effect using small electrical currents (< 1–2 mA) via anodal or cathodal stimulation [103, 104]. Some authors have shown that DCS, especially at the spinal level, may be a promising therapeutic strategy that can be used alone or in combination with other effective treatments to alleviate neuropathic pain [105].

To date, it is difficult to determine which has the greater efficacy in neuropathic pain in SCI.

tsDCS may involve long-term potentiation and long-term depression mechanisms and mediate changes in glutamatergic neurotransmission at the spinal level [106].

Peripheral sensitisation induced by tsTDCS triggers central sensitisation by spinal projection to supraspinal structures via ascending pathways [107]. In contrast, tDCS treatment has been shown to normalise the effects of central sensitisation by acting on the descending pain modulatory network [108]. Wrigley et al. proposed that the long injury duration may explain why tDCS did not work [68]. They hypothesized that an alteration in central pain transmission circuits after SCI becomes consolidated over a long period of time; therefore, tDCS may not be able to modulate the central pain-related system in chronic SCI, thus reducing its analgesic properties [68].

Yeh et al., in 2021, investigated the effects of multiple sessions of tDCS followed by moderate upper body exercise on neuropathic pain and brain activity in people with chronic SCI [67]. Multiple sessions of anodal tDCS combined with moderate upper body exercise were feasible for people with neuropathic pain after SCI. However, the analgesic effect was not superior to exercise alone after 12 sessions of intervention, and the beneficial effect was observed after 4 weeks of follow-up.

Among non-invasive brain stimulation techniques, repetitive transcranial magnetic stimulation (TMS) plays a role as a potential treatment option for neuropathic pain, particularly in cases where conventional treatments are ineffective [44].

TMS has been approved by the US Food and Drug Administration (FDA) for the treatment of several central nervous system (CNS) disorders, including chronic pain [45]. This technique uses electromagnetic coils to generate a magnetic field that modulates neuronal excitability, potentially relieving pain by influencing different brain regions [45]. The therapeutic effect of rTMS in neuropathic pain is primarily attributed to its ability to modulate cortical excitability and neuroplasticity [109]. rTMS delivers focused magnetic pulses that create electrical currents in targeted brain regions, thereby influencing neuronal firing patterns and synaptic connections. With repeated application, rTMS can produce lasting changes in the neural networks associated with pain perception [110].

As tDCS, the primary target of TMS for chronic neuropathic pain is the primary motor cortex (M1), with the most effective outcomes achieved through high-frequency (5–20 Hz) lateral stimulation at 80% of the motor threshold [44, 111].

Daily high-frequency M1 TMS has also been shown to be well tolerated and to provide temporary pain relief in patients with neuropathic pain and combining rTMS with pharmacotherapy and physical rehabilitation may further enhance and sustain pain relief. [112].

rTMS has significant potential as a non-invasive, safe, and effective treatment option for SCI patients. By targeting specific brain areas and applying the principles of neuroplasticity, rTMS can provide meaningful pain relief and improve quality of life.

rTMS appears to alter dysfunctional pain pathways in SCI patients, using frequency-specific effects to either inhibit or excite neural networks involved in pain processing. High frequency rTMS (≥ 10 Hz) generally increases cortical excitability, whereas low frequency stimulation (≤ 1 Hz) inhibits it, allowing tailored approaches to pain management [113].

However, the optimal TMS treatment protocols and the mechanisms by which TMS affect brain function and neuropathic pain in SCI patients remain unclear, limiting its wider clinical application in this population.

Research suggests that TMS-induced pain relief may involve remodeling of the endogenous opioid system, restoration of normal cortical excitability and inhibition of nociceptive signaling [111]. As tDCS, TMS might also regulate the expression of inflammatory markers [111]. One study found that active excitatory M1 TMS promoted pain recovery during the transition from acute to chronic pain [114], while others showed that high-frequency M1 TMS administered at 3-week intervals produced sustained analgesia, supporting its potential for treating refractory chronic pain [115].

The 2013 study by Jetté et al. provides evidence for the short-term analgesic effects of rTMS by testing the effects of a single session of high-frequency (10 Hz) rTMS targeting primary motor cortex (M1) areas associated with hand and leg motor function. The results showed that a single rTMS session targeting either motor area significantly reduced pain within the first 48 h after stimulation. This finding suggests that even a single rTMS application can provide short-term pain relief, which may be clinically valuable for immediate pain management. Interestingly, patients with incomplete lesions experienced greater analgesic effects, suggesting that residual motor or sensory pathways may enhance rTMS responsiveness, possibly due to greater cortical plasticity or preserved neurophysiological connections [116].

Sun et al. in 2019 suggested that high frequency rTMS, applied daily for six weeks in 17 patients, may be a promising adjunctive treatment for pain management in patients with SCI. Both the rTMS and sham groups experienced a gradual reduction in pain over the course of the study, but the rTMS group showed a more substantial analgesic effect, especially after two weeks of treatment.

The authors suggest that rTMS may help alleviate hypersensitivity in M1 and premotor cortex, which may be a mechanism for pain relief [63].

The 2017 study by Nardone et al. is consistent with Sun's 2019 findings in supporting the analgesic benefits of rTMS. However, there are notable differences in stimulation targets and protocols that provide additional insight into how rTMS might best be applied in SCI-related pain management.

Nardone et al. targeted both the prefrontal cortex (PMC/DLPFC), which is associated with pain processing and emotional regulation, and measured both sensory and affective dimensions of pain. The results showed that, although the sample size was very small, active rTMS significantly reduced both sensory and affective components of pain compared to baseline, highlighting the potential of targeting prefrontal regions to address not only the sensory but also the emotional burden of neuropathic pain in SCI [43].

Furthermore, the findings of Jetté et al. in the context of the longer protocols of Sun and Nardone suggest that while single rTMS sessions may provide short-term relief, repeated sessions (as in Sun's study) may be necessary to achieve sustained pain reduction.

Zhao et al. (2020) provide an important perspective on the potential of rTMS by focusing on pain in the early post-SCI period (3 months), comparing the effects of rTMS over the hand motor cortex with a sham group while both groups received standard physical, occupational, and medical therapies.

There was a significant reduction in pain scores in the rTMS group, suggesting that rTMS, when applied soon after SCI, may offer an early intervention strategy to prevent chronic pain, potentially reducing long-term central sensitization or maladaptive plasticity [69].

In contrast, in their study, Defrin and colleagues investigated the effects of a 10-session rTMS protocol on chronic central pain in paraplegic SCI patients. Both the real and sham rTMS groups reported significant reductions in pain right after each session, but the lack of long-term differences between the groups suggests a strong placebo or procedure effect.

The findings of Defrin et al. underscore that while rTMS may provide short-term relief of central SCI pain, lasting effects may be difficult to achieve without prolonged or repeated treatment, especially in cases of long-standing pain. The use of the vertex as the site of stimulation, rather than a more targeted motor area, may also play a role in these results [117].

Yilmaz et al. found no significant long-term difference in pain reduction between the real and sham rTMS groups. Both groups experienced a transient reduction in pain intensity, with the real rTMS group showing pain relief at 10 days and 6 weeks, whereas the sham group showed pain relief only at 10 days. However, by 6 months, neither group had sustained pain reduction, suggesting that rTMS did not provide a superior benefit over placebo for long-term pain management in this setting.

The difference with other studies may be due to the study duration and treatment protocol with a limited duration that may not have provided sufficient stimulation to produce lasting neural changes, the placebo effect, and the patient population and duration of chronic pain [64].

Kang and colleagues in 2009 investigated the effects of a 5-day rTMS protocol targeting the hand motor cortex for chronic neuropathic pain in SCI. Their results showed no significant difference between the real and sham rTMS treatments in pain reduction or interference with daily activities, indicating that this protocol did not produce therapeutic effects.

This study is consistent with the findings of Yilmaz and Defrin, who also reported no sustained benefit of rTMS over sham for chronic SCI pain. The lack of efficacy here may be due to the short duration of 5 days, which may be insufficient to induce lasting neuroplastic changes in chronic pain pathways. In addition, the heterogeneity of SCI levels and pain sites (e.g., trunk, pelvis, limbs) may affect the uniformity of rTMS effects, as different pain sites may respond differently to motor cortex stimulation [118].

These results suggest that short courses of rTMS may not be robust enough for chronic, widespread pain relief in SCI and further support the need to explore longer or tailored protocols targeting specific pain sites for more effective analgesia.

The mechanisms underlying increased cortical excitability differ between tDCS and rTMS. tDCS generates a weak, continuous electric current, whereas rTMS (and epidural stimulation) delivers repetitive pulses of electric current at near-threshold intensities. tDCS is thought to alter the electrical properties of the neuronal membrane, resulting in either hyperpolarization or depolarization of the targeted area [119, 120]. In contrast, rTMS induces synaptic plasticity through long-term potentiation and depression [119]. An important difference between these techniques is that tDCS tends to have a more widespread effect and a longer lasting effect compared to rTMS [121].

However, both methods can produce similar indirect changes in the activity of connected brain regions that may influence pain perception.

For pain control, the optimal site for rTMS stimulation is not the painful area itself, but a nearby region [44].

In contrast, epidural stimulation is most effective when electrodes are placed according to the cortical somatotopic representation of pain [64].

With tDCS, due to the large size of the electrodes, stimulation is likely to modulate a larger area of the motor cortex, affecting both the painful region and adjacent areas. While the degree of pain relief may vary between these brain stimulation techniques, previous research suggests that patients who respond to epidural stimulation also tend to benefit from non-invasive methods such as rTMS [122].

Furthermore, thalamic activity changes have been observed following motor cortex stimulation with both TMS [123] and tDCS [124]. The large electrode size in tDCS likely causes excitability modulation over a wide area centered on the motor cortex, including regions representing the painful body parts. This may explain why tDCS applied to the motor cortex is effective in treating neuropathic pain affecting multiple areas, such as the lower limbs and trunk [125].

While further replication of these findings is needed, if confirmed, they would support the idea that non-invasive stimulation techniques such as tDCS and rTMS produce lasting neurophysiological effects that could be beneficial for treating conditions such as chronic pain that are associated with cortical dysfunction.

Among such non-invasive brain stimulation techniques, TBS, a protocol that involves shorter bursts of patterned stimulation, has been also shown to produce robust effects [126]. TBS is a form of TMS that applies a series of rapid pulses of magnetic stimulation to the brain [46]. The "theta burst" refers to the pattern of stimulation, which mimics the natural theta rhythm in the brain (around 4–7 Hz), a frequency associated with light sleep, relaxation, and certain types of memory processing [46]. TBS is typically delivered in short bursts (bursts of 3 pulses at 50 Hz, repeated at a frequency of 5 Hz, or 3 pulses every 200 ms) for a brief period, making it much faster than traditional TMS protocols. There are two main types of theta burst stimulation: Continuous Theta Burst Stimulation (cTBS) which involves a continuous series of theta bursts that is typically associated with inhibitory effects on the brain area being stimulated (reducing neuronal activity); Intermittent Theta Burst Stimulation (iTBS) which involves bursts of theta rhythm interspersed with brief pauses that is usually associated with excitatory effects on the brain (increasing neuronal activity) [46]. TBS has attracted interest in the treatment of SCI-related pain due to its potential to modulate neural activity and promote neuroplasticity. On this basis, Gharooni et al. in 2018 suggested that TBS may help alleviate neuropathic pain by targeting specific brain regions associated with pain perception and processing. Stimulation may lead to long-lasting changes in synaptic strength, potentially reducing pain signaling pathways. In this regard, the Gharooni study has shown positive results with intermittent theta burst stimulation (iTBS) over the primary motor cortex for upper limb sensorimotor dysfunction following spinal cord injury [65].

Finally, other techniques have been studied to demonstrate their efficacy on SCI-related pain management, such as visual illusion (VI) associated to TENS.

VI therapy is an emerging technology that uses specialized hardware and software to create realistic or synthetic three-dimensional (3D) environments, enabling immersive interaction within the virtual environment [127]. In the field of SCI-related pain management, VI may help reducing pain perception, possibly preventing maladaptive plasticity, thereby promoting neuroplasticity [128]. It likely works by enhancing critical mechanisms, such as the sensorimotor gating of pain [129], and by activating a network of brain regions referred to as the mirror system [130].

TENS, on the other hand, is a non-invasive method that sends low-voltage electrical currents through the skin to stimulate nerves, which can reduce pain perception [131].

Combining these approaches could yield interesting results, as they both target brain processing of pain. In this regard, Ozkul et al., in 2015 considered the effects VI and TENS treatments on twenty-four SCI patients with neuropathic pain [61], showing, immediately after the application of TENS and VI, a significant decrease in pain intensity in SCI patients. Considering that both treatment methods reduce pain intensity in short periods, they could be helpful and promising to ensure rapid effects when SCI-related neuropathic pain is most severe.

Furthermore, despite being a systematic review of RCTs, the modest quality of the included studies prevents the authors from drawing clear conclusions regarding the comparative efficacy of the different proposed techniques to manage SCI-related neuropathic pain. Moreover, another study limitation is the lack of data on neuropathic pain, that could be also assessed by specific scales (e.g., Neuropathic Pain Scale, Douleur Neuropathique 4 Questions, etc.). Lastly, the total number of subjects in each treatment modality remains relatively small to draw strong conclusions.

## Conclusions

Taken together, findings of this systematic review and meta-analysis showed that rehabilitative approaches seemed to be effective to relieve SCI-related pain. However, to date, the choice of which one could represent the gold standard is still under debate. Nonetheless, the current lack of high-quality trials calls for further research to address key issues regarding the optimal therapeutic protocols. Lastly, further insights are needed to better identify and profile SCI patients with neuropathic pain, categorized by demographic features, clinical severity, medications, disease onset and duration, outcome measures and concomitant conventional or other rehabilitation approaches.

## Data Availability

All data generated or analyzed during this study are included in this article. Further inquiries can be directed at the corresponding author.
